# Fluorescence-Labeled Amyloid Beta Monomer: A Molecular Dynamical Study

**DOI:** 10.3390/molecules25153524

**Published:** 2020-08-01

**Authors:** János Gera, Gábor Paragi

**Affiliations:** 1 Department of Medical Chemistry, University of Szeged, H-6720 Dóm square 8, 6720 Szeged, Hungary; gera.janos@med.u-szeged.hu; 2 MTA-SZTE Biomimetic Systems Research Group, Department of Medical Chemistry, University of Szeged, H-6720 Dóm square 8, 6720 Szeged, Hungary; 3 Institute of Physics, University of Pecs, H-7624 Ifjusag utja 6, 7624 Pecs, Hungary

**Keywords:** amyloid beta (1-42) monomer, replica exchange molecular dynamics, fluorescent dye, Alexa 568, conformation analyses

## Abstract

The aggregation process of the Amyloidβ (Aβ) peptide is one of the central questions in Alzheimers’s research. Fluorescence-labeled single-molecule detection is a novel technique concerning the early stage investigation of Aβ aggregation, where the labeling dyes are covalently bound to the Aβ monomer. As the influence of the dye on the conformational space of the Aβ monomer can be significant, its effect on the seeding process is an open question. The applied fluorescent molecule continuously switches between an active (ON) and an inactive (OFF) state, where the latter supports an extra rotational restriction at many commercially available dyes. However, only a few theoretical studies simulated the Aβ monomer in the presence of a dye and none of them considered the difference between the ON and the OFF states. Therefore, we examined the impact of a selected fluorescence dye (Alexa 568) on the conformational space of the monomeric Aβ(1–42) peptide in its ON and OFF state by replica exchange molecular dynamic simulations. Investigations on secondary structure elements as well as dye-peptide contact analysis for the monomers are presented. Experimental and theoretical NMR shifts were contrasted to qualify the calculation protocol and theoretical values of the labeled and the non-labeled peptide were also compared. We found that the first five residues have higher helical propensity in the presence of the dye, and electrostatic properties could strongly affect the connection between the dye and the peptide parts.

## 1. Introduction

Alzheimer’s disease (AD) is one of the most common types of neurodegenerative disorder in the elderly population [1,2]. Pathologically, AD can be characterized by the accumulation and aggregation of amyloid β (Aβ) peptides in the central nervous system. Aβ peptide is cut out from the Amyloid Precursor Protein by β and γ secretases resulting in a fragment made up of between 39 and 43 residues. Recent studies have shown that there is a structural difference between fibrils seeded in vitro from Aβ monomers and samples derived from patients [3,4]. The mechanism of fibril formation is not trivial and extremely difficult to characterize experimentally due to the heterogeneity and transient nature of the initial oligomers [5,6]. Nowadays, it is widely accepted that early stage oligomerization could be more toxic than mature fibrils due to the interaction with different synaptic receptors [7,8]. Therefore, information about the background of the seed formation of Aβ peptides should be useful for the understanding of the aggregation process and can help us to identify critical points in the early stage mechanisms.

Concerning the starting steps in the assembling of Aβ monomers, NMR measurement was the only experimental technique for a long time, which could gain insight into the structural character of Aβ peptides [9,10]. The conclusion of these studies was that the Aβ peptide was mostly intrinsically disordered (IDP) and did not form a stable conformation. The most recent solution NMR study [9] pointed out that chemical shifts and J couplings of Aβ40 and Aβ42 isoforms hardly differ from the random coil structure, as well as that there is no difference in these values concerning the two monomeric peptides comparing residues 1–34. This finding suggests that both peptides are predominantly disordered, provide very similar conformational space, and the population of compact secondary structures (like β-hairpins) in the central hydrophobic core region (CHC) is low.

Nevertheless, the application of NMR in the investigation of the early stage assembling of the Aβ peptide proved to be challenging because of the high aggregation propensity of the monomer. However, a number of new techniques have been gaining ground in the past 10 years, such as fluorescently-labeled single-molecule detection, and have begun to be applied in the early phase of Aβ aggregation investigations [11,12]. The great advantage of single molecule spectroscopy over the solution NMR technique is that the experimental concentration of the monomer can be so low (100 pM or below) that in practice there is no chance for it to form larger aggregates or fibrils and even the detection of the monomeric state can be achieved by observation of a single photobleaching step of the dyes attached to the peptide. Furthermore, there is no need to perform experiments at low temperatures to slow down the aggregation process, which is a widely applied technique in NMR measurement. Thus, the crucial question regarding advanced single-molecule detection techniques is how the direct labeling can affect the conformational space of the Aβ monomer. It is also known that fluorescence dyes switching between an ON- and OFF-state in advanced super-resolution spectroscopic measurements, such as (direct) stochastically optical reconstruction microscopy (fluorescence), photoactivated localization microscopy or stimulated emission depletion microscopy [13]. The principal difference between the ON- and OFF-states is that the latter one contains an extra intramolecular bond at certain cases (e.g., dyes from Alexa or ATTO families) which affect the rotational degree of freedom of the fluorophore part. More precisely, it fixes the relative orientation within the dye of the fluorophore part and the connecting benzoic acid ring (see Figures S1 and S2 in the supplementary information). In case of a flexible molecule such as the Aβ monomer the two states can affect the conformational space of the labelled molecule differently, but this difference was never investigated before by computational simulation.

Molecular dynamics (MD) calculations can provide an atomic level interpretation of conformational variability and it can give a valuable supplement to experimental investigations [14]. It supports the structural characterization of disordered peptides, as the experimental determination of these features proved to be a challenging task. Concerning geometrical descriptors like secondary structure elements or contact maps, it is important to note that the outcomes of the analysis of computationally generated conformational space were found to be rather heterogeneous [15,16,17,18,19] and the success seems to heavily depend on the selected method [20,21,22]. Recently, Head-Gordon and her colleagues [23] pointed out that a properly selected advanced sampling technique, such as a Replica Exchange Molecular Dynamics (REMD) simulation, is a crucial step regarding the quality of the calculation. Moreover, several studies have suggested that particular force fields are more suitable to reproduce local NMR observables [24,25], thus careful selection of the force field or the water model is also important.

Interestingly, in spite of the fact that the Aβ peptide is the subject of numerous computational studies, there are very few MD simulations of the direct labelled Aβ peptide [26,27] Although, advanced experimental fluorescence imaging techniques usually apply an Aβ peptide fragment with direct labeling (see [12] and references therein), according to our knowledge, the first full length computational study was the work of H. S. Chung et al. [9,27]. In this work, experimental and computational simulations were applied together, and the *N*- as well as the *C*-terminal part were covalently labelled by dyes of 40 and 42 units long Aβ peptides. They investigated the Förster resonance energy transfer efficiency differences regarding the two monomers, which related to the distance between the covalently attached dyes at the terminals. Therefore, they did not examine the conformational space difference between labelled and non-labelled Aβ peptide. Having a guess about the effect of covalently attached dyes on the conformational space of small peptides we would like to refer to the work of M. Zacharias et al. [28] or M. Sauer et al. [29].

Taking into account all these facts, we aimed to compare the conformation space of labeled and non-labeled Aβ(1-42) monomers by REMD simulations. The selected labeling dye was the commercially available Alexa568 with its factory linkage as a routinely applied fluorescent dye in recent super-resolution techniques. Moreover, we aimed to examine not only the fluorescently active state of the dye, but OFF-state conformation analyses were also performed, where the specific form of the two states are presented in the supplementary information (Figures S1 and S2).

## 2. Results

### 2.1. Cα Chemical Shifts

**Figure 1:**
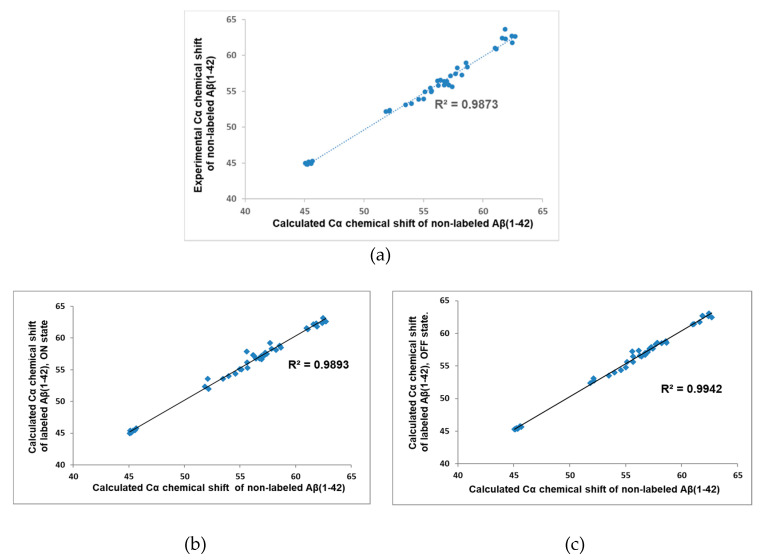
(**a**) Comparison of experimental and calculated Cα chemical shifts taking the non-labeled Aβ(1-42). Comparison of calculated labeled Cα chemical shifts of Aβ(1-42) peptide in the ON-state (**b**) and in the OFF-state (**c**).

Cα atoms play a central role in the recognition of secondary structure elements; therefore, their NMR chemical shift is often the subject of experimental or computational investigation. A good correlation between experimental and theoretical Cα chemical shifts for peptides and proteins is an indication for the proper calculation method. For the corroboration of the selected force field and the explicit water model, experimental and computational Cα chemical shifts were compared first considering the non-labeled monomeric peptide. Experimental results were applied from Roche et al. [9], while theoretical chemical shifts values were evaluated from cluster representatives (clustered by the Cα atoms of the peptide). The cluster representatives were selected to cover at least 90% of the total conformation space and the weighted average of the representative’s values are presented in Figure 1A where the weighting factors were proportional to the size of the associated clusters. The calculated Cα chemical shifts were in good agreement with the experimental values (R^2^ = 0.9873); therefore, the Amber98sb-ILDN force field parameter with TIP3P explicit water model and REMD protocol seem to be suitable to sampling the conformational space.

In the next step, we examined whether the presence of the dye could modify the Cα chemical shifts. So, Cα chemical shifts were calculated for the Alexa 568 labeled peptides in both states, and we compared them to the non-labeled calculated values, as experimental values for labeled peptide were not available. We found a strong correlation (R^2^ = 0.9893, 0.9942) between the labeled and the non-labeled cases, as it clearly showed in Figure 1B,C.

So, our calculation method provided excellent agreement with the experimental results and the presence of the dye does not significantly affect the chemical shift. This latter statement implies that we cannot expect substantial change in the IDP character of the peptide either.

### 2.2. Secondary Structure Elements

Focusing now on the secondary structure elements, the collapsed coil structure was suggested by most recent studies as a possible conformation for the monomeric Aβ peptide [14,18,19,20,22]. According to that, the peptide did not adopt any strict secondary structure and in a dynamic ensemble the random coil dominates, which complemented with low propensity of other secondary structure elements. Small molecules as well as peptide mimetics can modify the secondary structure profile of the peptide [30,31,32], thus it is reasonable to assume that covalently bonded molecular dyes can affect the conformational ensemble of the monomer.

**Figure 2:**
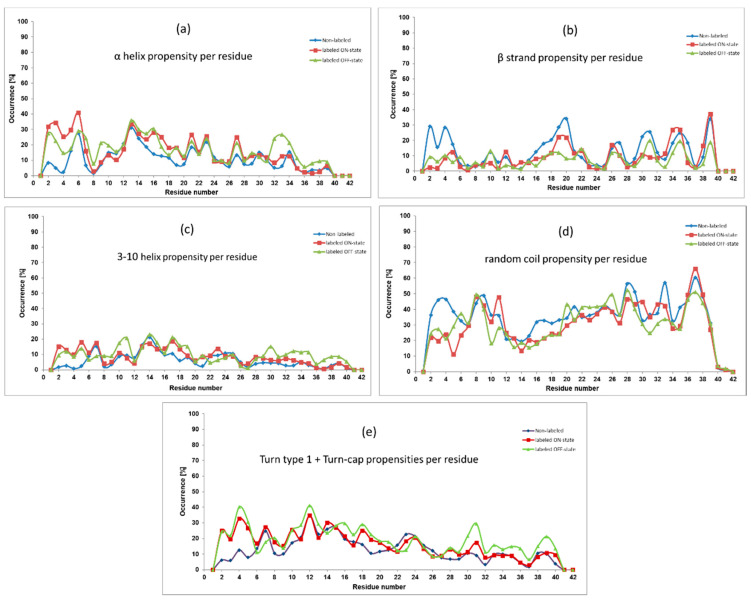
Occurrence (in %) of α helix (**a**), β strand (**b**), 3–10 helix (**c**), random coil (**d**) and turn types (**e**) secondary structure propensities for each residue.

Several secondary structure propensities were investigated, and the most significant ones are presented in Figure 2 in all the three cases (non-labeled and labeled in the ON/OFF states). On a large scale, the labeled peptides follow similar trends to the non-labeled one, but certain differences can also be mentioned.

Salient alterations can be observed in the *N*-terminal region (residues 2–5), where the presence of Alexa 568 increased both helical propensities and turn types propensity. Namely, amino acid residues of the labeled peptides showed 20–30% larger α helical and turn type propensities as well as 10–20% higher 3–10 helix occurrence than the non-labeled monomer. We would like to mention that according to the DISICL classification of secondary structure elements [33], the 3–10 helix content, the Turn 1 type and the Turn Cap elements together constitute the so-called 3–10 helical turn. For clarity, we presented the 3–10 helix content and the turns content separately. On the other hand, the non-labeled Aβ peptide showed higher random coil (in average 22.6% and 16.3%) and β-strand (in average 16.3% and 14.7%) propensities comparing to the ON and OFF states, respectively.

Considering the central hydrophobic core (CHC) region (residues 16–21), which has an important role in the fibril stabilization, the β-strand occurrence changed between 5–35%. Comparing labeled and non-labeled monomers, the latter one had always higher β-strand propensity and the difference was the largest at residues Phe19 and Phe20. Regarding helices, in the presence of the dye higher helical occurrence could be found. However, the differences between propensity values of the non-labeled and the labeled peptides were smaller in this region than at the previously mentioned *N*-terminal part.

Focusing now on the residues in the second half of the peptide, we could mentioned the increased turns propensity (which is the constituent of the 3–10 helical turn element) in the OFF state at residues 30–39. Interestingly, none of the other secondary structure elements were decreased in the other cases. Finally, regarding the full sequence, the OFF-state peptide showed the largest turns propensity in general, while it seemed that the largest β-strand content was achieved by the non-labeled peptide.

### 2.3. Interacting Residues with the Fluorescent Part of the Dye

**Figure 3:**
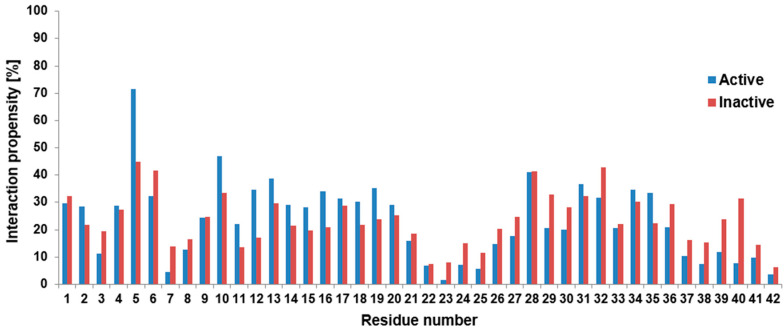
Interaction propensity per residue according to the close contact of the fluorophore part of the dye and the concerned residue.

In the case of monomers, the formation of secondary structure elements is usually related to intramolecular contacts; therefore, the effect of the dye can be also examined by the investigation of the contact between the original Aβ peptide and the fluorophore part of the dye. The fluorophore part contains five connecting rings with an extended delocalized electronic system, as well as several heteroatoms or charged chemical groups (see the structure in the supplementary information), whose chemical variability supports the ability of numerous secondary interactions. On the other hand, it is not always trivial to distinguish the chemical origin of a close contact, therefore, simply the contact distribution between the fluorophore part of the dye and all the residues within the peptide were calculated and presented in Figure 3 in its active (ON) and inactive (OFF) states.

It is clear from Figure 3 that on a large scale both states followed similar tendencies, again. So, the rotational constraint in the OFF state did not induce a large difference. However, we can also see from Figure 3 that at the first half of the peptide, the active state had slightly more frequent contact with amino acids, especially between residues 10 and 20. This region includes the CHC part, which has an important role in the seed formation of the small Aβ oligomers.

It is also clearly seen at residues 7,8 as well as at residues 22–25 that there was a local minimum in the contacts; namely interaction propensities were around 10% or below. Since at these positions we could find negatively charged residues, these minima could be the consequence of electrostatic repulsion. This explanation can be reinforced by the situation at residue 11: it is a negatively charged glutamic acid, which, due to the electrostatic repulsion, could prefer fewer contacts with the dye locally than its adjacent neutral hydrophobic neighbors. Therefore, residue 11 had lower contact than the adjacent residues. Furthermore, the most stable connection was formed between the active fluorophore part and Arg5, but the connection in the inactive state was also significant at this residue. Checking this proximity, we found a strong interaction between the SO_3_(−) group at the dye and the positively charged guanidino group from the arginine. This connection is a good example of why we focused simply on the geometrical contacts: it is hard to distinguish in this case, whether it is a hydrogen bond connection, or whether we should handle it as an electrostatic zwitterionic interaction. Nevertheless, we analyzed the intramolecular hydrogen bonds along the trajectories between the fluorophore part and the peptide. According to this investigation, the Arg5 residue had the largest number of hydrogen bond connections with the dye. It is worth mentioning that at residue 28 (Lys28) we could also find a local maximum in both cases. Since Lys28 was a positively charged residue in the calculations, this increase could be the consequence of an electrostatic attraction, as well.

According to Figure 3, there was, again, increased contact between residues 29–36. However, this region is known, again, for its strong hydrophobic character and some regions of it usually form hydrophobic connections with the CHC part, according to previous theoretical or experimental investigations (see the review of [34] and references therein). Because of the hydrophobic character of the fluorophore part of the dye it could have the same kind of connection with this region similar to the CHC part.

### 2.4. Free Energy Landscape (FEL) and Aggragation Propensities

**Figure 4:**
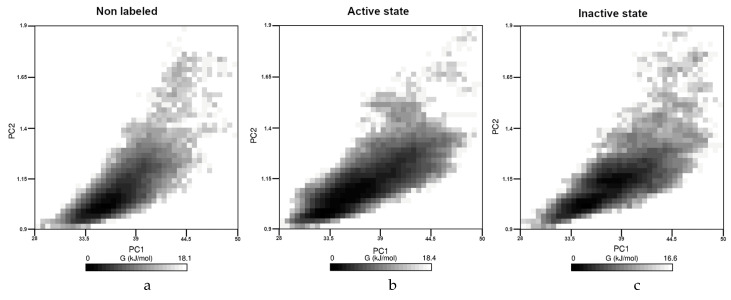
The Free Energy Landscape of the non-labeled (**a**), labeled ON (**b**) and labeled OFF (**c**) state. PC1 was always the SASA value (in nm^2^) and PC2 was the RoG (in nm). Free energy was presented in kJ/mol.

Finally, aggregation propensities and FELs were also determined for all the three case. From a chemical point of view, the change in the solvent accessible surface area (SASA) or in the Radius of Gyration (RoG) can be considered as quantities that can correlate with the aggregation propensity [35]. Therefore, these values were calculated, and the results with statistical descriptors (minimum-maximum values without outliers, median and interquartile range) were presented in the supporting information. Here, the FEL are presented in Figure 4 for all the three cases.

As we can see, the character of the three figures was very similar. In all cases the SASA and the RoG values were in the same range (28 nm^2^ to 50 nm^2^ or 0.9 nm to 1.9 nm, respectively), and the calculated energy was changed very similarly between 0 and 18.4 kJ/mol. We could not detect any separated region; therefore, we think that the presence of the dye cannot affect drastically the aggregation propensity of the monomer. The statistical descriptors of RoG and SASA values along the trajectories presented in the supporting information (Figure S3) strengthen these results.

## 3. Discussion

In the present study we investigated the effect of covalently bounded fluorescence dye molecules on the conformal space of the 42 units long monomeric Aβ peptide. Since the conformational freedom of the dye depends on its active (ON) or inactive (OFF) state, independent calculations were performed for both states, as well as for the non-labeled original Aβ monomer. This latter calculation allowed us to check the quality of our simulation. Comparing the experimental Cα NMR shift results of the non-labeled monomer [9] to the calculated ones, we had 0.9873 R^2^ value (see Figure 1A), which showed an excellent correlation between the theoretical and the experimental results. Therefore, we think that the theoretically generated conformational ensembles properly sampled the conformational space of the monomeric Aβ peptide. Then, we compared the calculated Cα NMR shifts of the non-labeled peptide to the calculated ones of the labeled peptides in both (ON/OFF) states. It showed very little difference (see Figure 1B,C), which forecasted a similar secondary structure content. Indeed, the investigation of the propensities of secondary structure elements (see Figure 2) showed very similar character on large scale, and they showed a collapsed coil structure according to the relatively high level of random coil elements at large parts of the peptide. However, a certain local effect could further refine the picture. In the *N*-terminal region (residues 1–5), the increase of the helical content in the presence of the dye happened at the expense of the other two structure elements, as we could see it clearly in Figure 2. Less characteristically, this was also the situation for residues 14–20, as well. Having an atomic level explanation on the origin of this findings, we examined the intramolecular contact between the amino acids of the peptide and the fluorophore part of the dye. As Figure 3 pointed out, the strongest contact between a residue and the fluorophore part was formed at residue Arg5. Here, a zwitter ionic or hydrogen bonded intramolecular connection was formed between the negatively charged SO_3_(−) group at the fluorophore part and the guanidino group from the arginine This can force the alteration of the original β strand and random coil structures. Having an idea about the manifestation of this contact we presented representative structures (Figure S4) in the supporting information to visualize the formation of the contact between SO_3_(−) group and Arg5.

Focusing now on further residues, we found very low contacts at Asp7, Glu22 or Asp23. It could be the consequence of an electrostatic repulsion between the dye and the amino acids, since all these residues were originally negatively charged. The importance of the electrostatic interaction was strengthened by the local decrease or increase in the contacts between the fluorophore part and Glu11 or Lys28. The increased contact number in the latter case also suggested that the low number of contacts in the Asp23 residue was not the consequence of the formation of the intramolecular salt bridge between Asp23 and Lys28. The Asp23-Lys28 connection has an important role in the fibril structure, therefore the formation of it could mean the steps toward the aggregation.

Furthermore, we also pointed out in Figure 3, that at the first half of the peptide, but especially at residues 10–20, the active state of the dye had a somewhat larger number of contacts than the inactive one. As this part of the peptide contains multiple hydrophobic regions (e.g., the LVFF sequence in the CHC region) the more flexible active state could more easily adopt the hydrophobic connection than the inactive state. It is worth noting, that in the inactive state the large delocalized electronic system in the fluorophore part is partially destroyed; however, this change in the molecular mechanical level calculations is less characteristic than in quantum mechanical investigations, where the electronic system is explicitly handled. It can raise the question of how this change can affect e.g., the hydrophobicity of the fluorophore part, but these investigations are beyond the scope of the present study.

Interestingly, the presence of the dye could affect another important connection in the aggregation path of the Aβ peptide, namely the hydrophobic sticking of the CHC part and a hydrophobic region at residues 32–36. These two regions are in strong hydrophobic contact in the fibrils, but according to Figure 3, both of them had extended interactions with the fluorophore part of the dye. These results could raise the question whether the presence of the dye could affect the kinematics of the Aβ aggregation, but monomeric investigations could not address this problem. Finally, the similarity of FEL as well as of RoG or SASA results suggested that the aggregation propensity of the peptides could not differ drastically in the presence of the dye, thus covalently attached dye should not disturb the investigation of early stage aggregation process.

## 4. Materials and Methods

The monomeric state of Aβ42 (PDB entry: 1IYT) was selected as the starting conformation. The peptide was measured by NMR in apolar microenvironment [10] being characterized by two helices between residues 8–25 and 28–39. The Alexa 568 was optimized first at the molecular mechanical level by the Polak-Ribier Conjugate Gradient method [36] using the Macromodel (Schrodinger, LLC., New York NY, USA) [37] program from the Schrodinger suite and then further optimized with Gaussian09 (Gaussian, Inc., Wallingford CT, USA) [38] at HF/6-31G** level of theory. For both states of the dye atomic charges were derived from the electrostatic potential fitting (ESP charges) and attached to the structure parameters with the Antechamber [39] program and other force constants of the dye were based on the GAFF parameter set [40]

Sampling the conformational space of monomeric Aβ42 peptide Replica Exchange Molecular Dynamics (REMD) simulations were carried out for all the three cases in explicit solvent using the GROMACS 2018-3 [41] package with the AMBER99SB-ILDN force field [42] for the Aβ42 peptide. A dodecahedron box (1.2 nm) was taken and solvated with TIP3P explicit water molecules.We would like to note here that according to the literature [24,25] this combination of force field and water model can provide a good agreement with available NMR data for the unlabeled monomeric peptide. Then, the system was neutralized with Na^+^ ions and additional Na^+^ and Cl^−^ ions were placed to imitate the physiological (0.15 M) salt concentration. All the complexes (Aβ42 and Aβ42 + dye) were energy minimized with 50,000 steepest-descent steps. Following the minimization process, the systems were heated up and 200 ps long NPT and NVT equilibrations were performed at 315 K. For REMD calculations, 48 replicas were selected in the range of 315 K and 400K based on the temperature distribution given by the web server of D. Van der Spoel [43]. All replicas were 250 ns long and temperature coupling was applied using velocity rescaling with a stochastic term [44] (0.1 ps time constant), an addition to the isotropic Parrinello-Rahman barostat [45] with 0.5 ps time constant. P-LINCS algorithm [46] was chosen for handling the hydrogen atom connection constrains while the electrostatic interactions were taken into account by the Particle Mesh Ewald (PME) method [47].

An ensemble analysis was performed in all the three cases using the lowest temperature (315 K) trajectories over the time interval between 100–250 ns. At this interval, each system reached its equilibrium, which can be seen according to the Cα RMSD diagrams presented in the supporting information (Figure S5). The Dihedral-based Segment Identification and Classification method (DISICL) [33] was selected to calculate the secondary structure elements in the conformation spaces. We applied the original definition in [33] of *φ*, ψ dihedral angles at the backbone for all the secondary structure elements.

Chemical shifts for the Cα were determined by SPARTA+ [48] program. The interacting residues were calculated over the selected time interval between each residue and the head group of the Alexa 568 dye, where contacts were defined as their center of mass distance should be lower than 0.4 nm. Hierarchical cluster analysis (single linkage method) was performed on the last 150 ns of the trajectory. For calculating the NMR values, those cluster middle structures were selected which were capable of representing 90% of the total conformational space. Chemical shifts for the Cα were determined by SPARTA+ [48] program.

## Supplementary Materials

The following are available online. Figure S1: The ON state of the Alexa568-Amyloid beta complex, Figure S2: The OFF state of the Alexa568-Amyloid beta complex. The blue circle shows the position of the extra bond in the OFF state, Figure S3: Summary of statistics regarding Radius of Gyration (left) and Solvent Accessible Surface Area (right) regarding the last 150 ns of the trajectory at T = 315 K replica. The RoG and SASA quantities were calculated in each frame along each trajectory and the most important statistical descriptors of the resulted distribution function (median, min-max, interquartile range) are presented for all the three systems. The dashed line shows the min, max values without outliers, the height of the box corresponds to the interquartile range and the line in the middle shows the median value, Figure S4: Snapshots to demonstrate the geometry of a close contact between SO3(-) and Arg5 (left) and between SO3(-) and Lys28 (right). The dye and its linkage were represented by the same color, Figure S5: The RMSD diagram of the Cα atoms along the full trajectories (0 ns–250 ns) in all the three cases.
