# Supplementary material for: Fluorescence-Labeled Amyloid Beta Monomer: A Molecular Dynamical Study
Source: Molecules. 2020 Aug 1;25(15):3524. doi: 10.3390/molecules25153524 (PMC7435871; doi:10.3390/molecules25153524)
Supplement: Supplementary file 1 [file molecules-25-03524-s001.pdf]

Fluorescence Labeled Amyloid Beta Monomer: A Molecular Dynamical Study

János Gera [1] and Gábor Paragi [2,3,*]

[1] Department of Medical Chemistry, University of Szeged, H-6720 Dóm square 8, Szeged, Hungary ; gera.janos@med.u-szeged.hu

[2] MTA-SZTE Biomimetic Systems Research Group, Department of Medical Chemistry, University of Szeged, H-6720 Dóm square 8, Szeged, Hungary ; paragi@sol.cc.u-szeged.hu

[3] Institute of Physics, University of Pecs, H-7624 Ifjusag utja 6, Pecs, Hungary; paragi@gamma.ttk.pte.hu

* Correspondence: paragi@sol.cc.u-szeged.hu, paragi@gamma.ttk.pte.hu ; Tel.: +36-62-544-4593

SUPPORTING INFORMATION

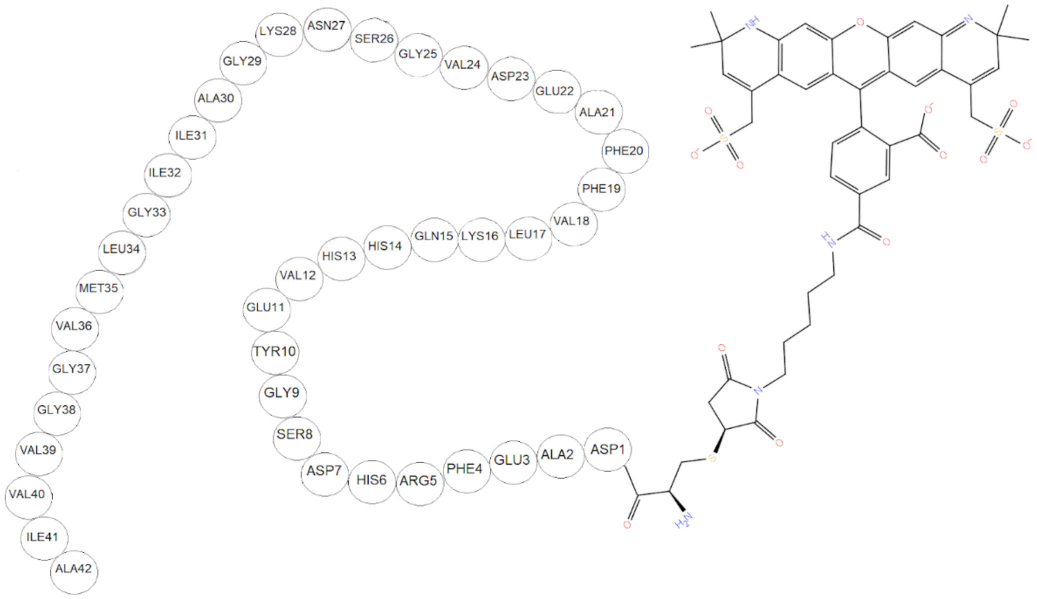

Figure S1: The ON state of the Alexa568 -Amyloid beta complex.

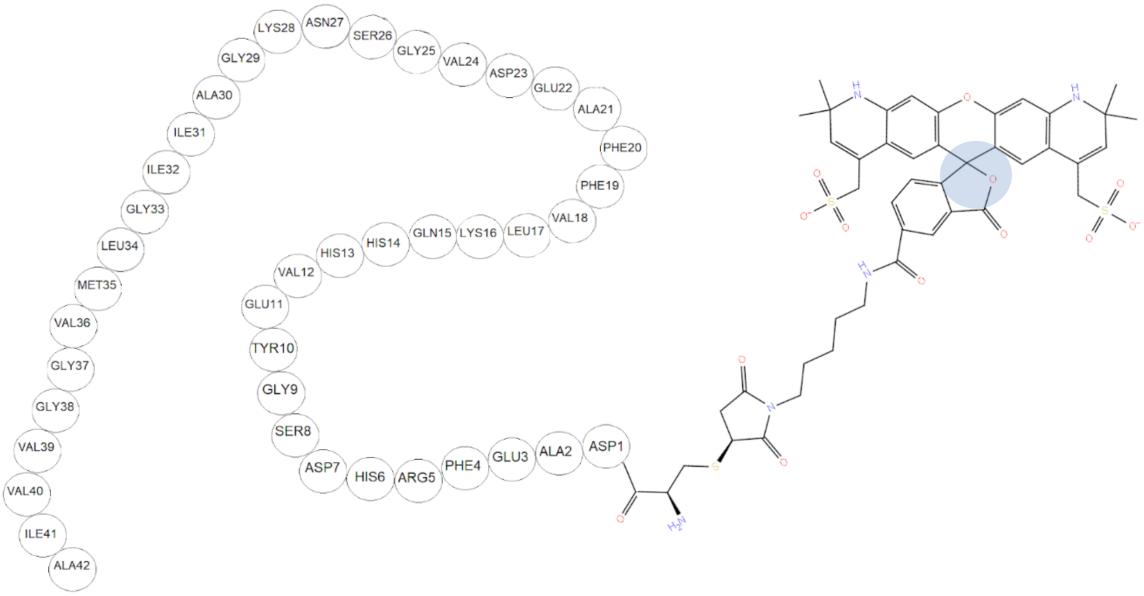

Figure S2: The OFF state of the Alexa568 -Amyloid beta complex. The blue circle shows the position of the extra bond in the OFF state.

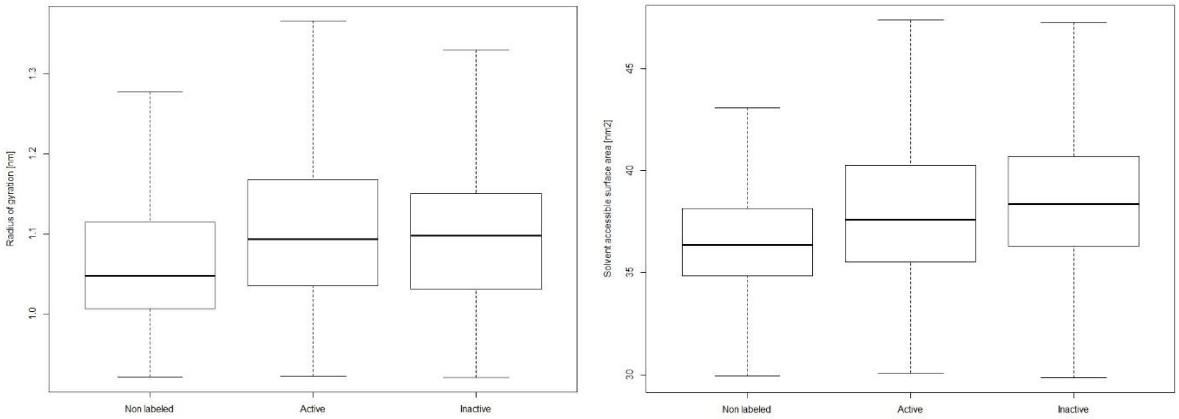

Figure S3: Summary of statistics regarding Radius of Gyration (left) and Solvent Accessible Surface Area (right) regarding the last 150ns of the trajectory at T=315K replica. The RoG and SASA quantities were calculated in each frame along each trajectory and the most important statistical descriptors of the resulted distribution function (median, min-max, interquartile range) are presented for all the three systems. The dashed line shows the min, max values without outliers, the height of the box corresponds to the interquartile range and the line in the middle shows the median value.

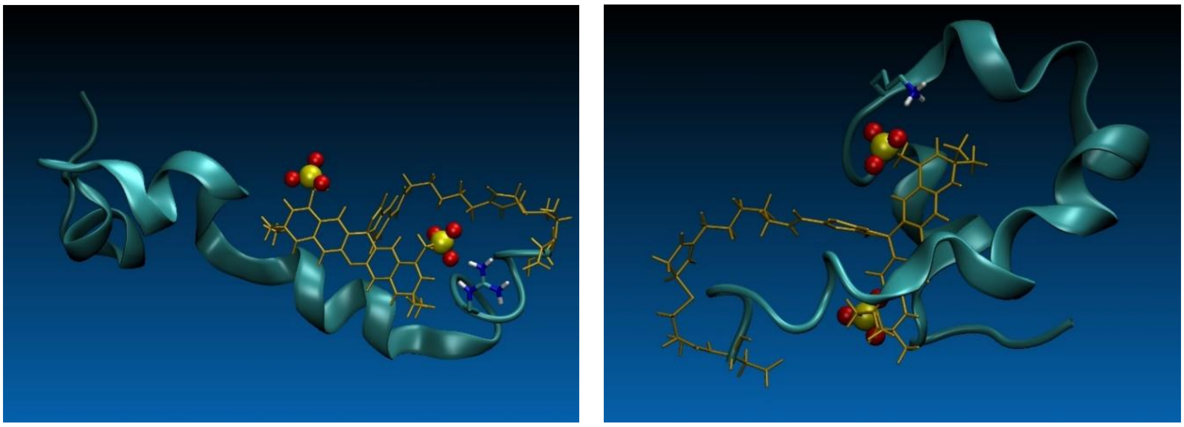

Figure S4: Snapshots to demonstrate the geometry of a close contact between SO_3(-) and Arg5 (left) and between SO_3(-) and Lys28 (right). The dye and its linkage were represented by the same color.

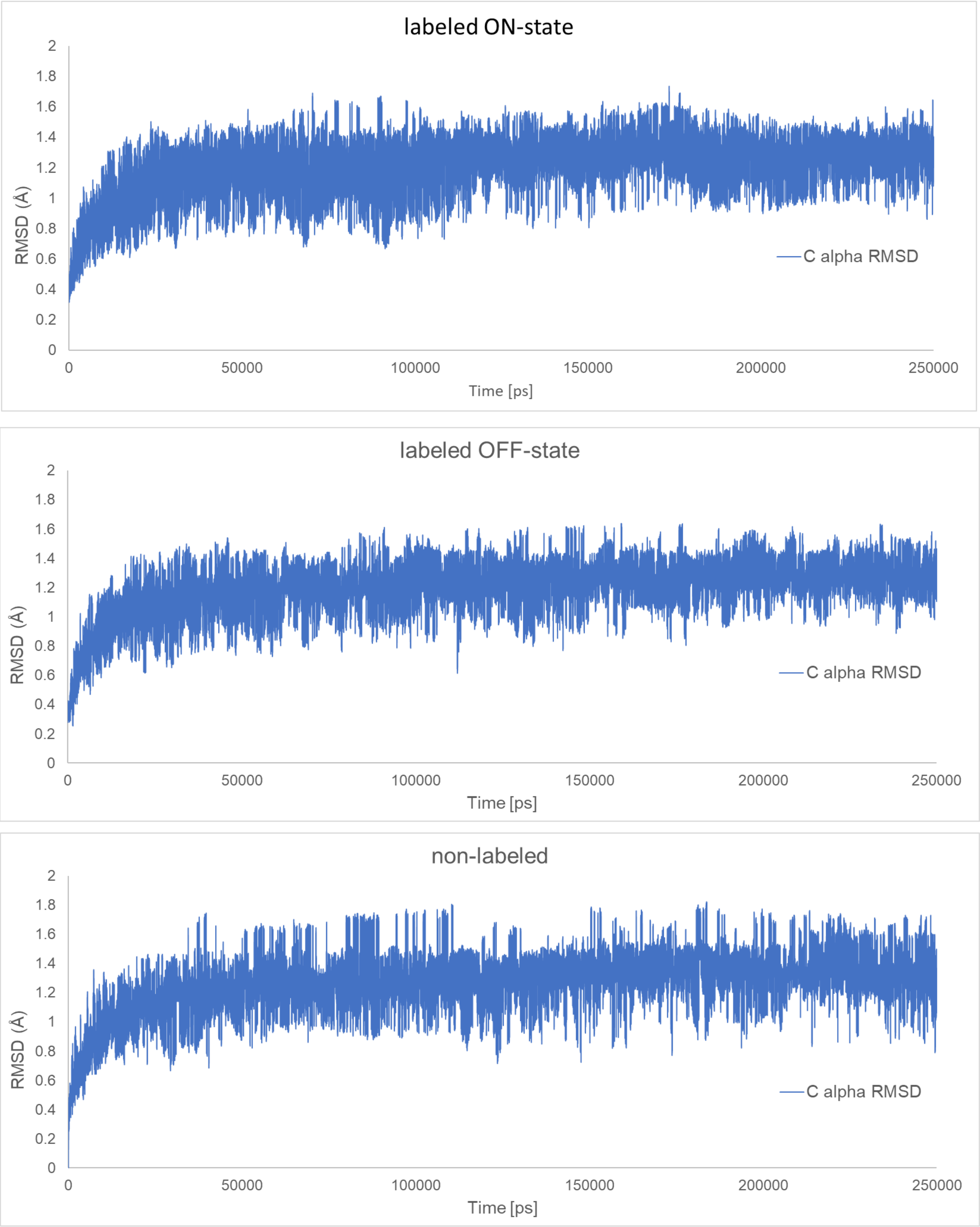

Figure S5 The RMSD diagram of the Cα atoms along the full trajectories (0ns-250ns) in all the three cases.
